# Metabolic phenotyping with computed tomography deep learning for metabolic syndrome, osteoporosis and sarcopenia predicts mortality in adults

**DOI:** 10.1002/jcsm.13487

**Published:** 2024-04-22

**Authors:** Sang Wouk Cho, Seungjin Baek, Sookyeong Han, Chang Oh Kim, Hyeon Chang Kim, Yumie Rhee, Namki Hong

**Affiliations:** ^1^ Department of Internal Medicine, Endocrine Research Institute Severance Hospital, Yonsei University College of Medicine Seoul South Korea; ^2^ Institue for Innovation in Digital Healthcare (IIDH) Yonsei University Health System Seoul South Korea; ^3^ Division of Geriatric Medicine, Department of Internal Medicine Yonsei University College of Medicine Seoul South Korea; ^4^ Department of Preventive Medicine Yonsei University College of Medicine Seoul South Korea

**Keywords:** computed tomography, metabolic syndrome, multi‐layer perceptron, osteoporosis, sarcopenia

## Abstract

**Background:**

Computed tomography (CT) body compositions reflect age‐related metabolic derangements. We aimed to develop a multi‐outcome deep learning model using CT multi‐level body composition parameters to detect metabolic syndrome (MS), osteoporosis and sarcopenia by identifying metabolic clusters simultaneously. We also investigated the prognostic value of metabolic phenotyping by CT model for long‐term mortality.

**Methods:**

The derivation set (*n* = 516; 75% train set, 25% internal test set) was constructed using age‐ and sex‐stratified random sampling from two community‐based cohorts. Data from participants in the individual health assessment programme (*n* = 380) were used as the external test set 1. Semi‐automatic quantification of body compositions at multiple levels of abdominal CT scans was performed to train a multi‐layer perceptron (MLP)‐based multi‐label classification model. External test set 2 to test the prognostic value of the model output for mortality was built using data from individuals who underwent abdominal CT in a tertiary‐level institution (*n* = 10 141).

**Results:**

The mean ages of the derivation and external sets were 62.8 and 59.7 years, respectively, without difference in sex distribution (women 50%) or body mass index (BMI; 23.9 kg/m^2^). Skeletal muscle density (SMD) and bone density (BD) showed a more linear decrement across age than skeletal muscle area. Alternatively, an increase in visceral fat area (VFA) was observed in both men and women. Hierarchical clustering based on multi‐level CT body composition parameters revealed three distinctive phenotype clusters: normal, MS and osteosarcopenia clusters. The L3 CT‐parameter‐based model, with or without clinical variables (age, sex and BMI), outperformed clinical model predictions of all outcomes (area under the receiver operating characteristic curve: MS, 0.76 vs. 0.55; osteoporosis, 0.90 vs. 0.79; sarcopenia, 0.85 vs. 0.81 in external test set 1; *P* < 0.05 for all). VFA contributed the most to the MS predictions, whereas SMD, BD and subcutaneous fat area were features of high importance for detecting osteoporosis and sarcopenia. In external test set 2 (mean age 63.5 years, women 79%; median follow‐up 4.9 years), a total of 907 individuals (8.9%) died during follow‐up. Among model‐predicted metabolic phenotypes, sarcopenia alone (adjusted hazard ratio [aHR] 1.55), MS + sarcopenia (aHR 1.65), osteoporosis + sarcopenia (aHR 1.83) and all three combined (aHR 1.87) remained robust predictors of mortality after adjustment for age, sex and comorbidities.

**Conclusions:**

A CT body composition‐based MLP model detected MS, osteoporosis and sarcopenia simultaneously in community‐dwelling and hospitalized adults. Metabolic phenotypes predicted by the CT MLP model were associated with long‐term mortality, independent of covariates.

## Introduction

Within an aging population, metabolism‐related disorders, including metabolic syndrome (MS), osteoporosis and sarcopenia, represent a burden on society due to their association with major complications such as cardiovascular events, fractures, morbidity and mortality.^1^, ^2^, ^3^ As these diseases are initially asymptomatic, early screening and diagnosis can lead to early intervention and lifestyle modifications, thereby preventing major adverse events. Therefore, appropriate screening is crucial, as pharmacotherapy can substantially reduce disability and death.^1^, ^4^

Changes in body composition, including muscle and visceral fat, are well known to be associated with aging. CT scans are commonly used imaging modalities, containing information on body compositions including skeletal muscle, fat and bone tissue. Due to recent advances in deep learning technology, computed tomography (CT)‐derived body composition parameters can be automatically quantified with better speed and accuracy, thus allowing such datasets to be used as quantitative imaging biomarkers.^4^, ^5^, ^6^ Several studies showed that quantified body composition features were proven to be useful to detect MS, osteoporosis and sarcopenia as separate models to detect each outcome.^6^, ^7^, ^8^

Based on these findings, we aimed to develop a multi‐outcome machine learning (ML) model that detects three common metabolic diseases (MS, osteoporosis and sarcopenia) simultaneously using CT body composition parameters. In addition, the prognostic value of metabolic phenotyping by a multi‐outcome ML model for long‐term mortality was investigated.

## Methods

### Study participants

#### Derivation cohort

**Figure 1:**
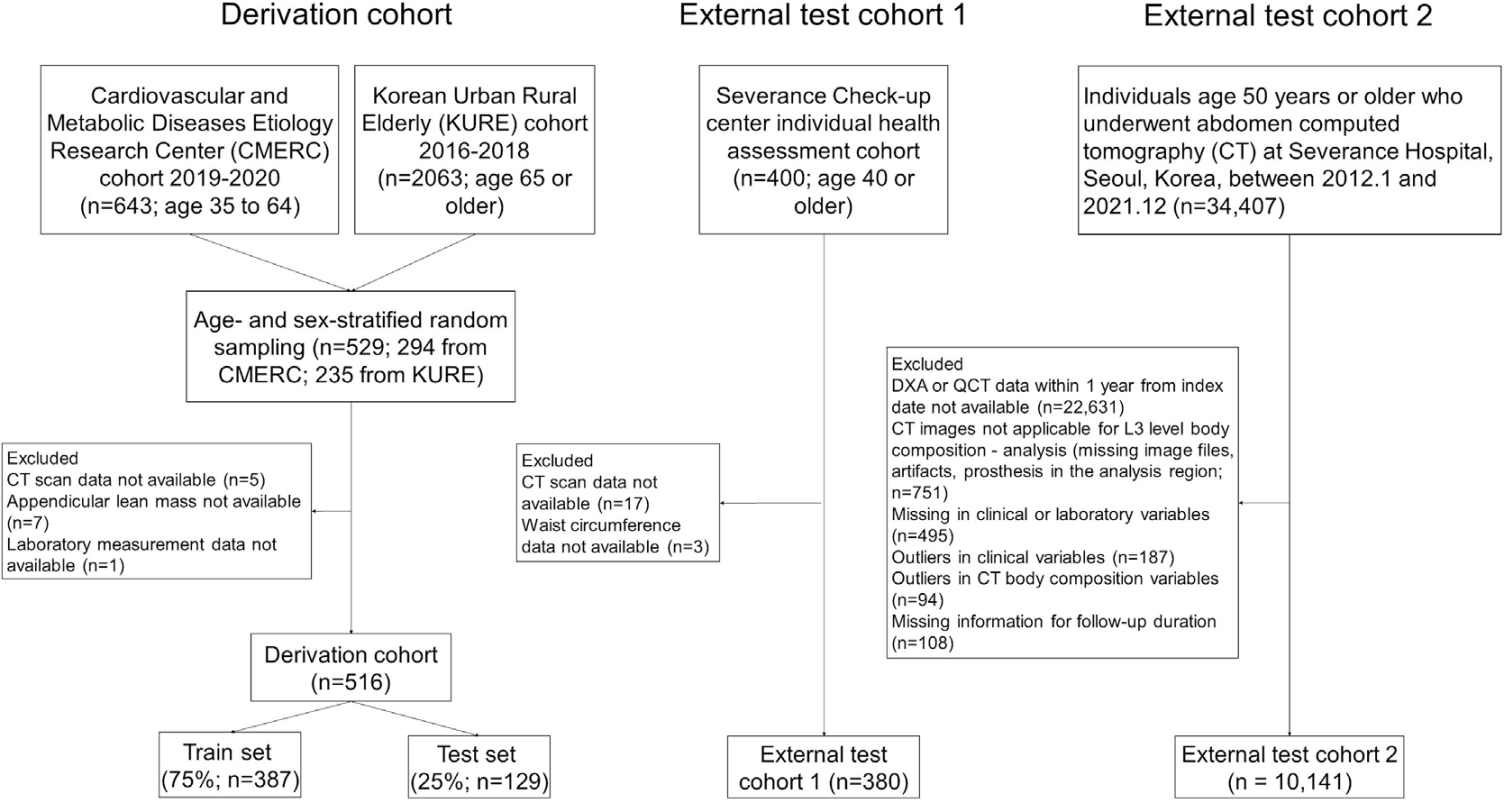
Study flow.

The derivation cohort consisted of two community‐dwelling adult cohorts: the Cardiovascular and Metabolic Diseases Etiology Research Center (CMERC) cohort and the Korean Urban Rural Elderly (KURE) cohort.^9^ CMERC was designed to identify risk factors for cardiac and metabolic diseases among community‐dwelling Korean adults aged 30–64, and the baseline evaluation was performed between December 2013 and March 2018. First‐wave follow‐up for CMERC participants who enrolled in 2013 was performed between 2019 and 2020, which included evaluation for sarcopenia features such as muscle function. The first wave of follow‐up CMERC data was used in this study. Detailed information regarding the CMERC cohort has been described in a prior paper.^9^ The KURE is a community‐based prospective, longitudinal cohort study on health in Korean older adults that began in 2012.^10^ The baseline assessment was conducted in three urban districts of Seoul and Gwanghwa, as well as a rural area of Incheon, between 2012 and 2015. The first wave of follow‐up for KURE was performed between 2016 and 2018, with a newly added investigation of whole‐body CT images. As this study required CT image analysis, data from the first wave of follow‐up for KURE were used. CMERC and KURE have been approved by the institutional review boards of Severance Hospital, Yonsei University Health System, Seoul, Korea (IRB Nos 4‐2013‐0661 and 4‐2012‐0172), and written consent for research was obtained from all study participants prior to the commencement of the study. For this analysis, the derivation set was constructed using age‐ and sex‐stratified random sampling from two cohorts. For stratified sampling, age was grouped by decades (six groups; 30–39, 40–49, 50–59, 60–69, 70–79 and 80 or older) for each sex; thus, a total of 12 strata were created. Up to 50 individuals were sampled for each stratum; if <50 were available for a group, all available datasets were included. As a result, a total of 529 individuals (294 from CMERC and 235 from KURE) were included (*Figure* *1* and *Table* *S1*). After excluding individuals with missing values in a CT scan, appendicular lean mass measurement or laboratory variables, 519 subjects remained in the final derivation cohort. The derivation cohort was then randomly split into the training set (75%) and the internal test set (25%).

#### External test cohort 1

External test cohort 1 was built to test the diagnostic performance of the CT‐based multi‐outcome (MS, osteoporosis and sarcopenia) multi‐layer perceptron (MLP) model trained in the derivation cohort. Data from individuals aged 40–79 who underwent health examinations at Severance Hospital Check‐up Center, Seoul, Korea, between November 2014 and December 2016 were used as the external test cohort.^11^ Participants were sampled by age (by decades) and sex strata, with up to 50 participants in a group (total *n* = 400; *Figure* *1* and *Table* *S1*). This study was approved by the Institutional Review Board of Severance Hospital, Korea, with a waiver for written informed consent for medical record reviews (IRB No. 4‐2017‐1115). After excluding individuals without analysing CT scans and waist circumference information, a total of 380 participants remained in the final external test cohort.

#### External test cohort 2

External test cohort 2 was built to evaluate the prognostic performance of the metabolic phenotyping by CT‐based multi‐outcome MLP model for predicting 10‐year mortality. Data of 34 407 individuals aged 50 or older who underwent unenhanced abdomen CT for any reason between January 2012 and December 2021 were reviewed (*Figure* *1*; IRB No. 4‐2021‐0771). After excluding those without evaluable CT scans, outliers in clinical values or missing values, a total of 10 141 individuals remained in external test cohort 2. The data of remained individuals were reviewed for occurrence of death and time to event between index date (the date when the CT was taken) and 31 December 2022, which was ascertained by reviewing the death certificate in the electronic record system of the institution. The last follow‐up dates for individuals were the death date, the last outpatient visit date, the discharge date within 10 years or 4000 days if the observation period exceeded 4000 days. Age, sex and Charlson comorbidity index (CCI)^12^ at the index date were collected.

### Outcome definitions

After retrieving clinical data and laboratory measurements from the Severance Clinical Research Analysis Portal with a medical record review, the presence of MS, osteoporosis and sarcopenia was defined. MS was defined according to the National Cholesterol Education Program (NCEP) III guideline, with an Asian threshold for waist circumference.^13^ The presence of three or more of the following components was considered as MS: (1) abdominal obesity (waist circumference: man >90 cm; woman >85 cm); (2) high blood pressure (>130/80 mmHg); (3) impaired fasting glucose (>100 mg/dL); (4) hypertriglyceridaemia (>150 mg/dL); and (5) low HDL‐cholesterol (man <40 mg/dL; woman <50 mg/dL). In the external test cohort, osteoporosis was defined as a dual‐energy X‐ray absorptiometry (DXA; Discovery W model, Hologic, USA)‐derived bone mineral density (BMD) T‐score of −2.5 or lower in the lumbar spine, femoral neck or total hip using the National Health and Nutrition Examination Survey (NHANES) III Caucasian young female reference according to the World Health Organization (WHO) guideline.^14^ BMD was assessed using quantitative computed tomography (QCT; Pro, Mindways Software, TX, USA) in the derivation cohort, and a lumbar spine volumetric BMD lower than 80 mg/cm^3^ was used to define osteoporosis.^15^ In the derivation cohort, sarcopenia was defined according to the Asian Working Group for Sarcopenia (AWGS) 2019 definition: low grip strength (<28 kg for men and <18 kg for women); low chair rise test performance (>12 s); and low appendicular lean mass by bioimpedance analysis (<7.0 kg/m^2^ for men and <5.7 kg/m^2^ for women). As the other dataset lacks variables for muscle strength and physical performance, a low appendicular lean mass was used to define sarcopenia in the external test cohort.^16^

### Computed tomography protocols and semi‐automated body composition analysis

Details regarding the CT scanners and protocols used for the derivation and external datasets are presented in *Table* *S2*. We restricted all analysis to unenhanced CT scans in this study. Segmentation and quantification of CT images were conducted using the commercial software DeepCatch (DeepCatch: FDA‐cleared, artificial intelligence software for whole body composition analysis [https://medicalip.com/DeepCatch; DeepCatch, Version 1.0.0.0, MEDICALIP Co., Ltd., Seoul, Korea; MFDS Approved, Free Sales]). DeepCatch uses deep learning algorithms to segment and quantify body composition parameters at the whole‐body level semi‐automatically. We obtained a good average dice similarity coefficient (92.3–99.3%) for skeletal muscle, abdominal visceral fat and subcutaneous fat masks compared with those obtained using manual drawings produced by radiology experts (gold standard).^5^ Skeletal muscle area (SMA), skeletal muscle density (SMD), visceral fat area (VFA), visceral fat density (VFD), subcutaneous fat area (SFA), subcutaneous fat density (SFD) and bone density (BD) were estimated at the level of lumbar spines 1–4 (L1–L4) and WHO‐defined average waist (AW) levels (middle level between the margin of the 12th rib and the iliac crest; *Figure* *S1*).

### Metabolic phenotype clustering using the computed tomography body composition parameters

Unsupervised agglomerative hierarchical cluster analysis was performed to derive the metabolic disease clusters based on multi‐level CT body composition parameters. Our model was optimized using centroid linkage, and we identified three optimal clusters. These clusters were determined by drawing a horizontal line through the middle section of the dendrogram.^17^

### Multi‐label deep learning model architecture

An MLP multi‐label classification model^18^ was trained to detect MS, osteoporosis and sarcopenia simultaneously. The MLP has multiple layers of nodes that are connected with weights and use a non‐linear activation function to linearly separate indistinguishable data. Pytorch,^19^ a deep learning framework, was employed to develop our models. The network used in this study is illustrated in *Figure* *S2*, which consists of an input layer, two fully connected hidden layers and an output layer. The model hyperparameters are presented in *Table* *S3*. CT body composition features at the L3 level alone (CT alone model) or combined with clinical features (age, sex and body mass index [BMI]; CT + clinical model) were used as input features for the model. The results of the three output nodes were predictive probabilities of the presence of MS, osteoporosis and sarcopenia. The network was trained by adjusting weights and biases and then optimized through a gradient descent algorithm.^20^ Loss function was a criterion that optimized the three diseases using the one‐versus‐all loss based on max entropy. Using a sigmoid function, the results of the final node ranged between 0 and 1, and the optimal points were calculated using the point closest to the corner in the ROC plane.^21^ The internal test set (the hold‐out set) from the derivation cohort and the external test cohort were used to assess the performance of the MLP models in classifying the presence of MS, osteoporosis and sarcopenia. SHapley Additive exPlanations (SHAP)^22^ summary plots were created to evaluate the relative contribution of body composition features to each outcome.

### Statistical analysis

Clinical characteristics and CT body composition parameters of study participants were compared using an independent two‐sample *t*‐test, one‐way analysis of variance with Bonferroni post hoc correction or a *χ* ^2^ test as appropriate. Fractional polynomial regression models were built to describe the age‐related trajectory of the CT body composition parameters in study participants using the Stata ‘twoway fpfit’ code. The area under the receiver operating characteristic curve (AUROC) of MLP models (clinical, CT alone or clinical + CT models) was compared using the DeLong method.^23^ In external test cohort 2, the Kaplan–Meier survival curve was plotted for mortality according to CT multi‐outcome MLP model scores for the presence of MS, osteoporosis and sarcopenia (highest tertile vs. other tertile groups). Univariate and multivariable Cox proportional hazard models were built to assess the associations between predicted disease probabilities and the risk of mortality, adjusting for age, sex and CCI. All variables did not violate the proportional hazard assumption. All statistical analyses were performed using Stata 16.1 (StataCorp, TX, USA) and the Python SciPy 1.8 library. *P*‐values lower than 0.05 were considered statistically significant.

## Results

### Clinical characteristics of study participants

The clinical characteristics of subjects from the derivation and external test sets are presented in *Table* *1*. The average ages in the derivation (women 52%) and external test cohorts (women 87%) were 62.8 and 59.7 years, respectively, with an average BMI of 23.9 kg/m^2^ in both cohorts. The prevalence of MS, osteoporosis and sarcopenia was 23%, 28% and 10% in the derivation cohort and 24%, 21% and 19% in the external test cohort, respectively.

**Table 1:** Clinical characteristics of study participants

	Derivation cohort (*n* = 516)		External test cohort 1 (*n* = 380)
	Train set (*n* = 388)	Test set (*n* = 128)	
Age, years	62.8 ± 14.7	62.8 ± 14.4	59.7 ± 11.1
Women, *n* (%)	198 (51)	66 (52)	190 (50)
BMI, kg/m^2^	23.9 ± 3.0	23.8 ± 2.9	23.8 ± 2.6
Metabolic syndrome, *n* (%)^a^	88 (23)	28 (22)	91 (24)
Metabolic syndrome components, *n* [IQR]	1 [1–2]	1 [1–2]	1 [1–2]
Waist circumference, cm	83.1 ± 9.2	81.7 ± 9.1	84.4 ± 8.1
Abdominal obesity, *n* (%)	127 (33)	36 (28)	104 (27)
Fasting plasma glucose, mg/dL	97.2 ± 18.4	98.2 ± 17.6	100.3 ± 23.0
Impaired fasting glucose, *n* (%)	122 (31)	42 (33)	136 (36)
Systolic BP, mmHg	124 ± 17	123 ± 18	125 ± 15
Diastolic BP, mmHg	73 ± 9	73 ± 9	79 ± 10
High blood pressure, *n* (%)	170 (43)	53 (41)	175 (46)
Triglyceride, mg/dL	108 [80–151]	102 [81–140]	87 [58–119]
High triglyceride, *n* (%)	99 (25)	29 (23)	61 (16)
HDL‐cholesterol, mg/dL	54 [45–62]	53 [45–60]	49 [42–59]
Low HDL‐cholesterol, *n* (%)	86 (22)	31 (24)	127 (33)
Sarcopenia, *n* (%)^b^	39 (10)	12 (9)	74 (19)
ALM index, kg/m^2^	6.9 ± 1.0	6.9 ± 1.0	6.9 ± 1.0
Low ALM, *n* (%)	58 (15)	23 (18)	74 (19)
Grip strength, kg	29.3 ± 9.5	28.7 ± 9.8	N/A
Chair rise test, s	8.9 [7.0–11.9]	9.4 [6.7–12.4]	N/A
Low muscle function, *n* (%)	120 (31)	43 (34)	N/A
Osteoporosis, *n* (%)^c^	108 (28)	35 (27)	78 (21)
QCT spine vBMD, mg/cm^3^	114 ± 47	114 ± 45	N/A
DXA T‐score			
Lumbar spine	N/A	N/A	−1.0 ± 1.5
Femoral neck	N/A	N/A	−1.2 ± 1.0
Total hip	N/A	N/A	0.6 ± 1.0

Abbreviations: ALM, appendicular lean mass; BMI, body mass index; BP, blood pressure; IQR, interquartile range; N/A, not available in dataset.

^a^ Metabolic syndrome was defined as the presence of three or more components according to the National Cholesterol Education Program/Adult Treatment Panel III guideline with a modified waist circumference threshold for Asians (≥90 cm in men and ≥85 cm in women).

^b^ Sarcopenia was defined as the presence of low appendicular lean mass with low muscle function (either low grip strength or low chair rise test performance). Because muscle function measurements were not available in the external test cohort, sarcopenia was defined based on low appendicular lean mass alone.

^c^ Osteoporosis was defined as a quantitative computed tomography (QCT)‐derived lumbar spine volumetric bone mineral density (vBMD) of 80 mg/cm^3^ or lower in the derivation cohort. In the external test cohort, osteoporosis was defined as a dual‐energy X‐ray absorptiometry (DXA) bone mineral density T‐score of −2.5 or lower in the lumbar spine, femoral neck or total hip.

### Age‐related trajectory of computed tomography body composition parameters

**Figure 2:**
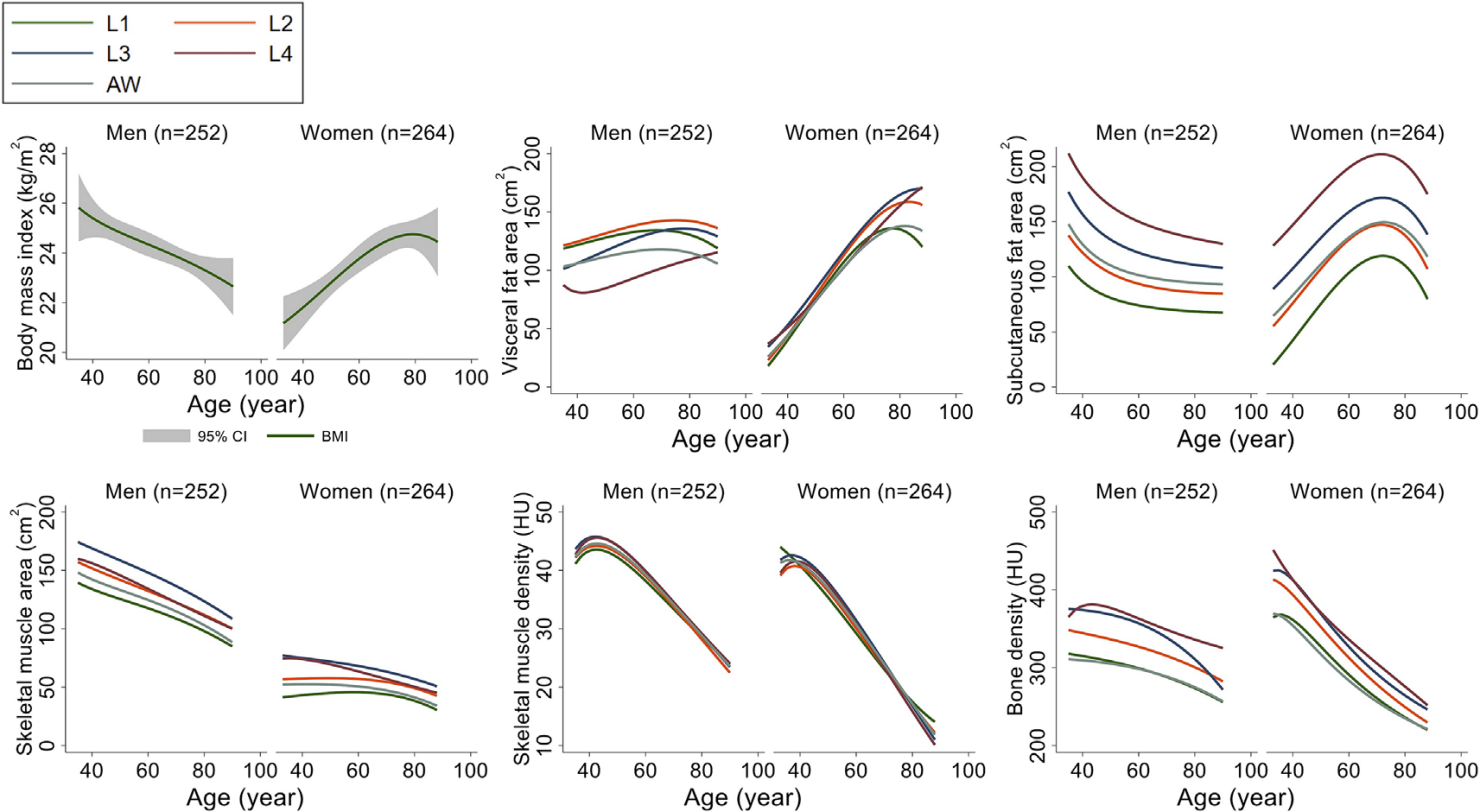
Age‐dependent trajectory of computed tomography‐derived body composition parameters in the derivation cohort. AW, average waist; HU, Hounsfield units.

The mean and standard deviation of CT body composition parameters at multiple levels are presented in *Table* *S4*. The age‐related trajectory of CT body composition parameters at multiple levels (L1–L4, AW) was visualized in the derivation set (*Figures* *2* and *S3*). A decrease in BMI was observed in men across age, whereas women showed an increase in BMI, along with a linear increase in VFA at all levels (L3 level) from age 30–39 to 80 or older (women: 33–119 cm^2^; men: 102–132 cm^2^). SFA showed a linear increase until 70 years of age in women, with a gradual decline afterwards. Among the different parameters, the L3 SMA showed the highest value in both men and women. SMA showed a decline across ages in both men and women, with a sharper decline in men. In women, the SMA plateaued until 70 years of age at most levels, with gradual decline afterwards (L3 level) from age 30–39 to 80 or older (women: 109–90 cm^2^; men: 170–120 cm^2^). However, unlike SMA, SMD showed a linear decrease in both men and women across age (L3 level) from age 30–39 to 80 or older (women: 43–17 Hounsfield units [HU]; men: 46–28 HU; 3.6–5.0 HU decrease/10 years), with similar values across measured levels (L1–L4). Women showed a sharper decline in BD compared with men, with the lowest values at the L1 level.

### Metabolic phenotyping with CT deep learning and mortality

**Figure 3:**
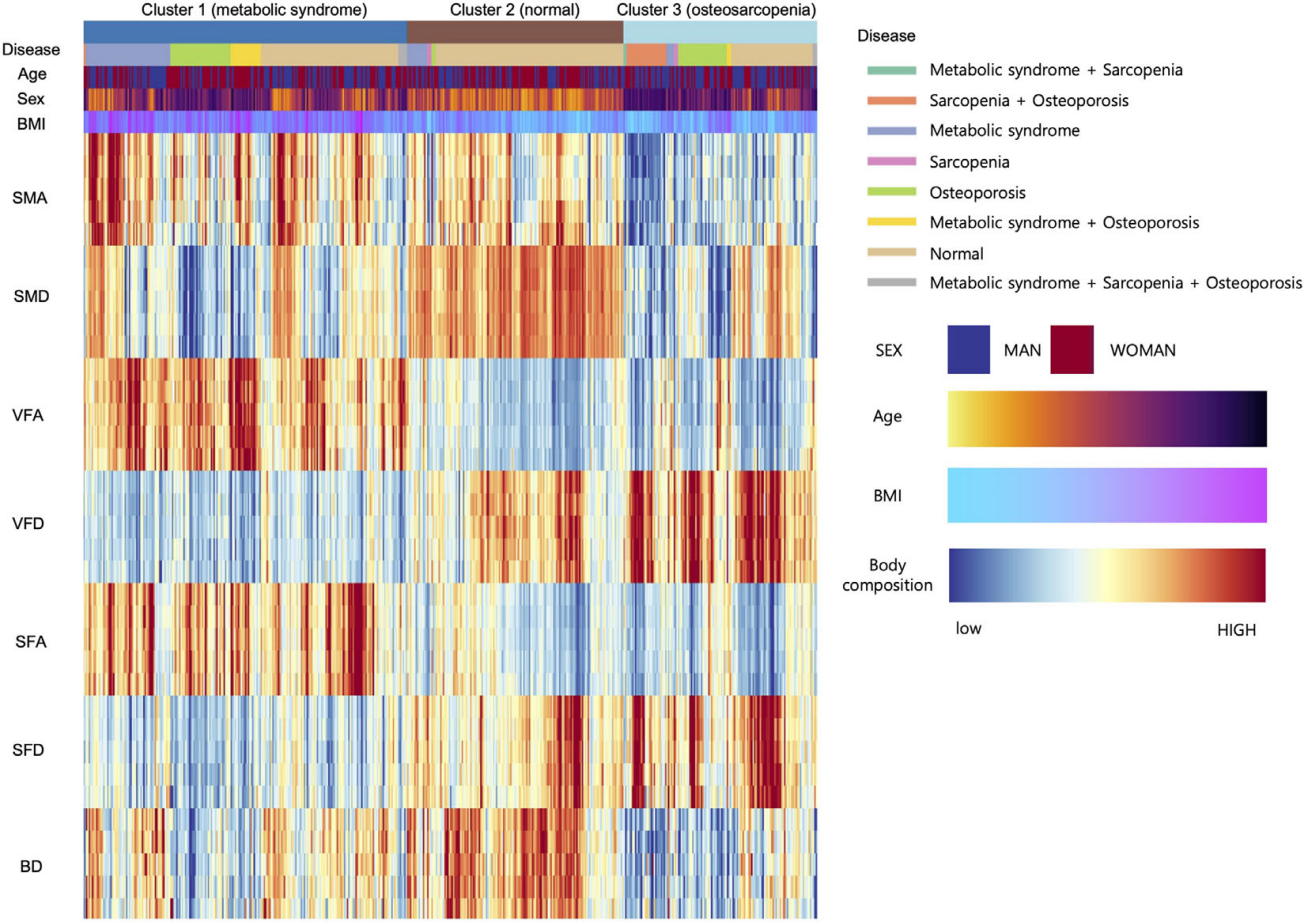
Computed tomography body composition‐based metabolic phenotype clusters obtained using unsupervised hierarchical clustering of the derivation cohort. BD, bone density; BMI, body mass index; SFA, subcutaneous fat area; SFD, subcutaneous fat density; SMA, skeletal muscle area; SMD, skeletal muscle density; VFA, visceral fat area; VFD, visceral fat density.

Hierarchical clustering was used to explore metabolic phenotype clusters based on CT body composition parameters. Body composition and clusters were visualized using a heatmap (*Figure* *3*). Three distinctive metabolic phenotype clusters were discovered: normal (*n* = 151), MS (*n* = 230) and osteosarcopenia clusters (*n* = 135). Clinical characteristics of metabolic phenotype clusters are presented in *Table* *S5*. Compared with the normal cluster, the MS cluster was associated with an older age (65 vs. 49 years), higher L3 VFA and SFA and lower SMD and BD values. The prevalence of MS in the MS cluster was highest among all clusters (39% vs. 9% in the normal cluster; vs. 10% in the osteosarcopenia cluster). The osteosarcopenia cluster had the oldest age among all clusters (72 years), with lower L3 SMA, SFA, SMD and BD values. The prevalence of osteoporosis and sarcopenia was 50% and 27%, respectively, in the osteosarcopenia cluster, being the highest among all clusters (2% for each in the normal cluster; 30% and 5% in the MS cluster, respectively; *Table* *S5*).

### Simultaneous metabolic phenotyping using the computed tomography body composition multi‐layer perceptron model

**Figure 4:**
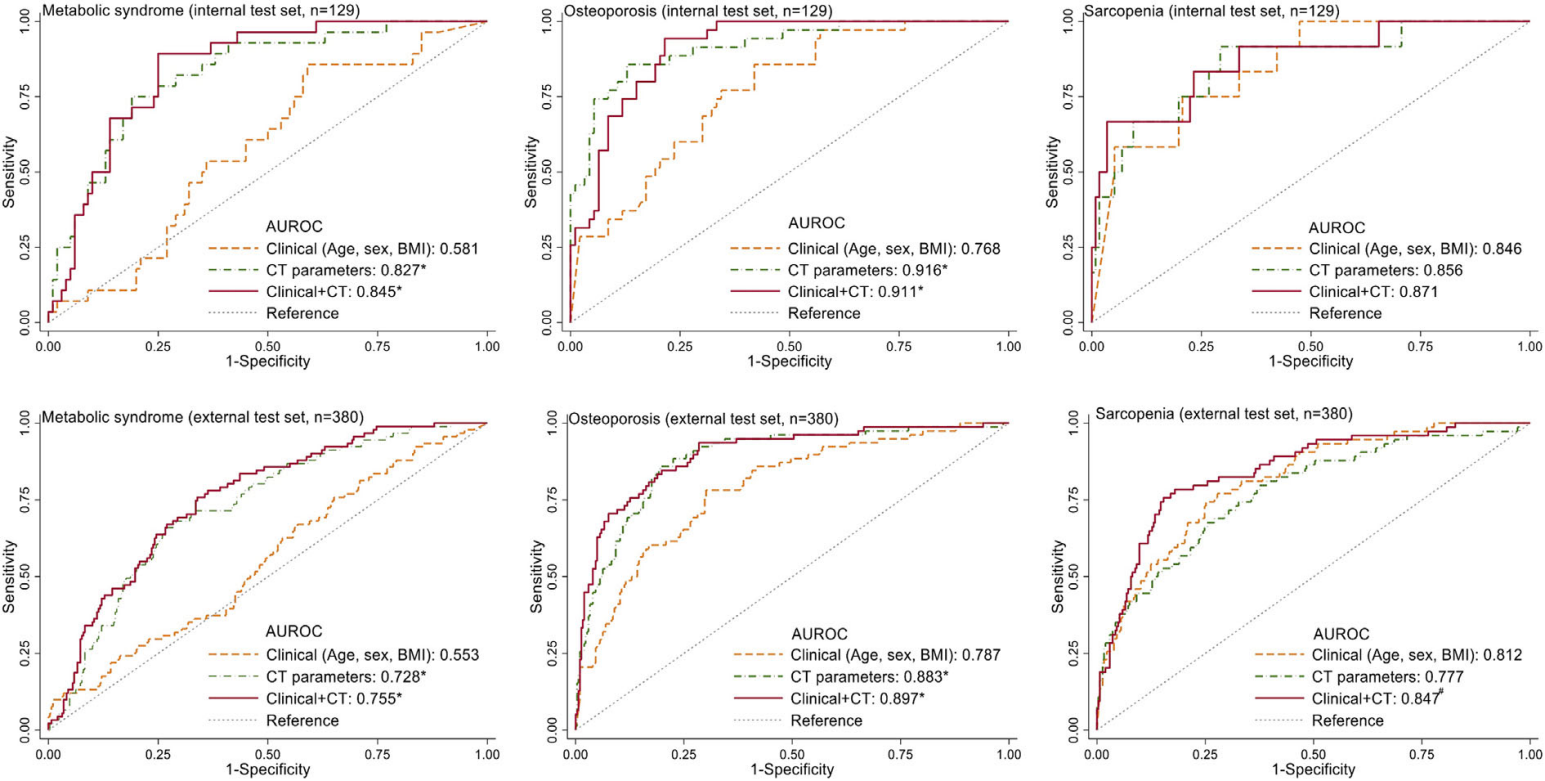
Comparison of discriminatory performance between clinical, computed tomography (CT)‐derived body composition parameters (L3 level) and combined models (CT + clinical) for the metabolic syndrome, osteoporosis and sarcopenia. Data represent the internal test set and external test set 1. AUROC, area under the receiver operating characteristic curve; BMI, body mass index.*P < 0.05 versus clinical model; ^#^ *P* < 0.05 versus CT parameter model.

**Figure 5:**
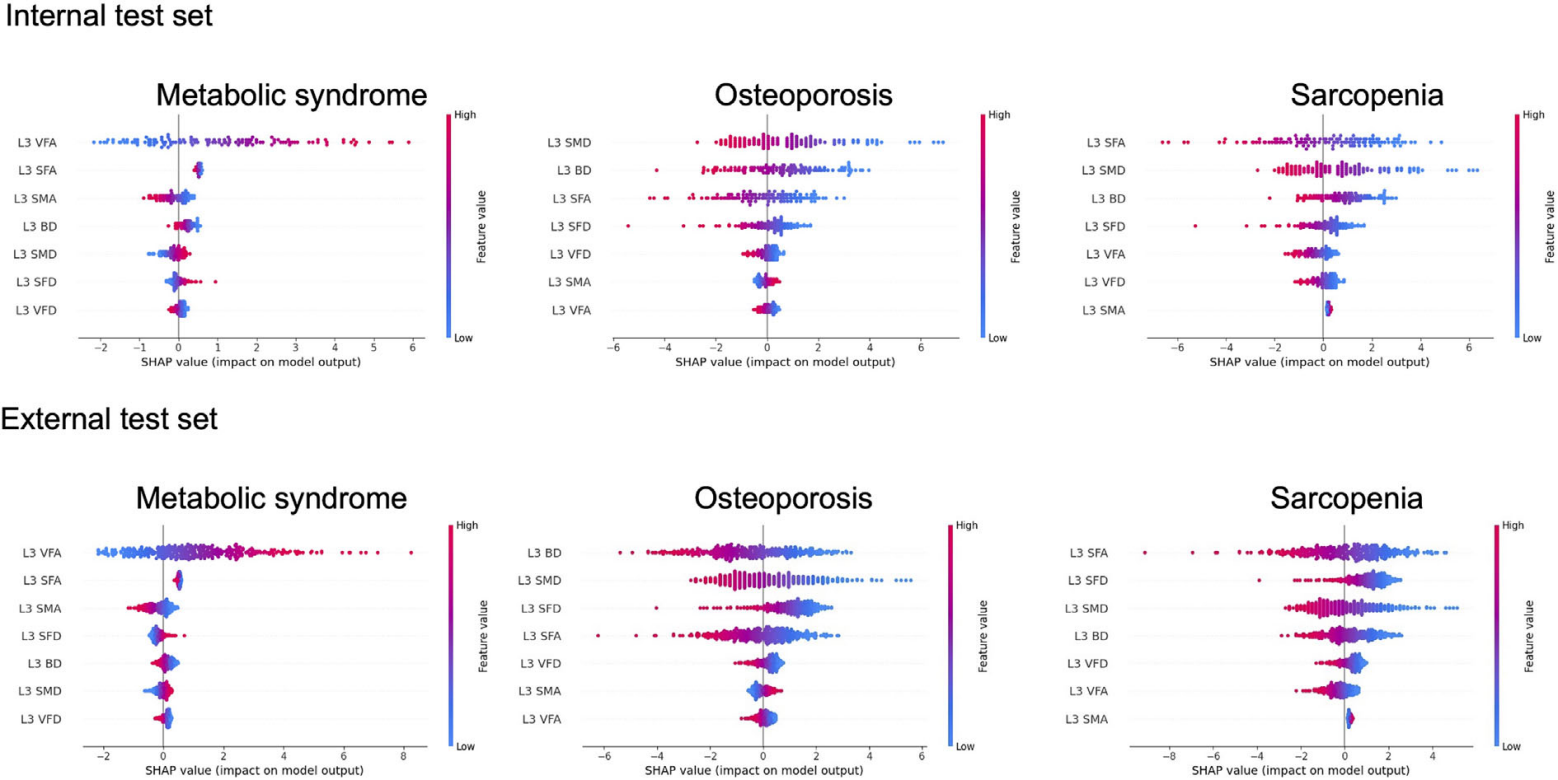
SHapley Additive exPlanations (SHAP) value of multi‐label multi‐layer perceptron models using computed tomography body composition parameters to predict the presence of metabolic syndrome, osteoporosis and sarcopenia in the internal test set and external test set 1. Body composition parameters were listed in descending order based on feature importance determined using the SHAP values.

An MLP model based on CT body composition parameters alone (CT alone model) was able to classify multiple metabolic diseases simultaneously, with good discriminatory performance in both internal and external test sets (AUROC: MS, 0.827 and 0.728 [for internal and external sets, respectively]; osteoporosis, 0.916 and 0.883 [for internal and external sets, respectively]; sarcopenia, 0.856 and 0.777 [for internal and external sets, respectively]; *Figure* *4* and *Table* *S6*). For the classification of MS and osteoporosis, both the CT alone model and the CT + clinical model outperformed the clinical alone model, whereas the performance of the CT alone and CT + clinical models did not differ significantly in both the internal and external test sets. For the classification of sarcopenia, the CT + clinical model outperformed the CT alone model (AUROC 0.847 vs. 0.777, *P* = 0.007) in the external test set. When the relative contributions of the CT body composition features on model outcomes were assessed using the SHAP plot (*Figure* *5*), the L3 VFA was the most important feature for determining the probability of the presence of MS (the higher the value, the higher the probability of MS) in both the internal and external sets. For osteoporosis, the L3 SMD was the most important contributing feature (the lower the value, the higher the probability of osteoporosis), followed by the L3 BD and the L3 SFA. The L3 SMD was also one of the important features contributing to the probability of sarcopenia presence, followed by the L3 SFA (the lower the value, the higher the probability), whereas the contribution of the L3 SMA was relatively small. When the CT alone or CT + clinical models were built using the CT body composition parameters obtained from different levels (L1, L2, L4 or AW) or all levels (using all CT body composition parameters obtained from L1 to L4 levels), a similar discriminatory performance was obtained for each outcome when compared with the results obtained using L3 models (*Figures* *S4* and *S5*).

### Computed tomography‐based metabolic phenotyping and long‐term mortality

**Figure 6:**
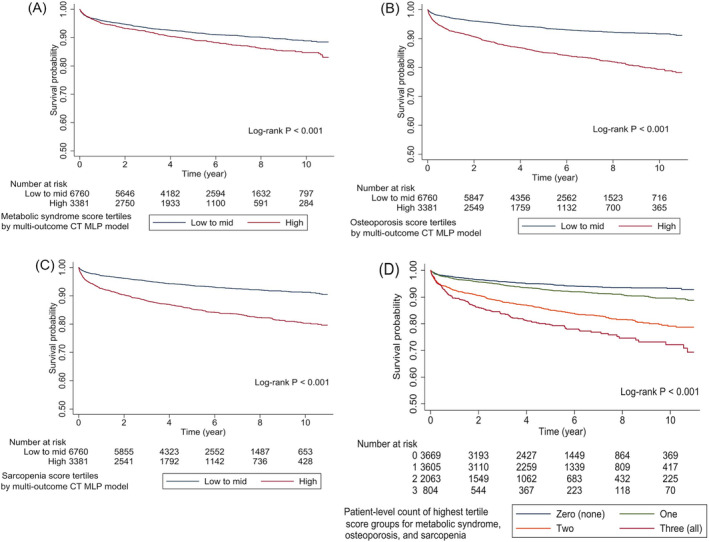
(A–D) Kaplan–Meier survival curve for mortality by computed tomography (CT) multi‐outcome multi‐layer perceptron (MLP) model scores for the presence of metabolic syndrome, osteoporosis and sarcopenia.

In external test cohort 2 (*n* = 10 141; mean age 63.5 years [range 50–97], women 79%; median follow‐up 4.9 years [range 0.9–10.1]), a total of 907 individuals (8.9%) died during follow‐up. The Kaplan–Meier survival curve was plotted for mortality according to CT multi‐outcome MLP model scores for the presence of MS, osteoporosis and sarcopenia (highest tertile vs. other tertile groups; *Figure* *6*). Compared with the referent (without any high tertile score groups), individuals with model‐derived high score tertiles for MS (*Figure* *6* *A*), osteoporosis (*Figure* *6* *B*) or sarcopenia (*Figure* *6* *C*) had a higher risk of mortality (log‐rank *P* < 0.001 for all). Individuals with combined metabolic phenotypes (presence of high CT MLP model score tertiles for two or more disorders) had a higher risk of mortality (two disorders combined: hazard ratio [HR] 3.02, 95% confidence interval [CI] 2.52–3.62; all three disorders combined: HR 4.31, 95% CI 3.48–5.33) compared with those with none (referent, HR 1.00) or single disorders (HR 1.41, 95% CI 1.17–1.70; *Figure* *6* *D*). In *Table* *2*, among various metabolic phenotypes, sarcopenia alone (adjusted HR [aHR] 1.55, 95% CI 1.18–2.03), MS + sarcopenia (aHR 1.65, 95% CI 1.14–2.40), osteoporosis + sarcopenia (aHR 1.83, 95% CI 1.46–2.29) and all three disorders combined (aHR 1.87, 95% CI 1.46–2.40) remained robust predictors of mortality after adjustment for age, sex and comorbidity index.

**Table 2:** Association of computed tomography multi‐outcome multi‐layer perceptron model‐detected metabolic phenotypes with 10‐year mortality

Metabolic phenotyping	Prevalence, *n* (%)	Death, %	Unadjusted model		Multivariable model (age, sex and CCI adjusted)	
			uHR (95% CI)	*P*	aHR (95% CI)	*P*
None	3669 (36)	5.3	Reference		Reference	
MS alone	1985 (20)	6.2	1.20 (0.95–1.50)	0.110	0.83 (0.65–1.04)	0.113
OS alone	729 (7)	9.5	1.82 (1.38–2.40)	<0.001	1.26 (0.95–1.68)	0.101
SC alone	891 (9)	8.3	1.56 (1.19–2.04)	0.001	1.55 (1.18–2.03)	0.001
MS + OS	377 (4)	11.9	2.43 (1.75–3.36)	<0.001	1.28 (0.90–1.80)	0.156
MS + SC	215 (2)	15.8	3.36 (2.33–4.84)	<0.001	1.65 (1.14–2.40)	0.008
OS + SC	1471 (14)	14.7	3.13 (2.58–3.81)	<0.001	1.83 (1.46–2.29)	<0.001
MS + OS + SC	804 (8)	18.9	4.31 (3.48–5.33)	<0.001	1.87 (1.46–2.40)	<0.001

Abbreviations: aHR, adjusted hazard ratio; CCI, Charlson comorbidity index; CI, confidence interval; MS, metabolic syndrome; OS, osteoporosis; SC, sarcopenia; uHR, unadjusted hazard ratio.

## Discussion

In this study, we discovered that a single multi‐label MLP model based on CT body composition parameters was able to classify MS, osteoporosis and sarcopenia simultaneously in community‐dwelling individuals. The age‐related trajectory of CT body composition parameters was observed, with a clear increase in fat components and a decrease in skeletal muscle mass, radiodensity and BD across the age span from 30s to 80s. Unsupervised clustering based on CT body composition parameters revealed three distinctive metabolic phenotype clusters: normal, MS and osteosarcopenia clusters.

The concept of utilizing CT body composition parameters as imaging biomarkers to detect metabolic disorders such as MS, osteoporosis or sarcopenia has been presented previously.^4^, ^6^, ^7^, ^8^, ^24^, ^25^, ^26^, ^27^ Pickhardt and colleagues showed that CT parameters, including total abdominal tissue and skeletal muscle index, yielded an AUROC of 0.916 for detecting MS (*Table* *S7*).^28^ Vadera et al. reported that the L1 radiation attenuation value was useful to discriminate the presence of osteoporosis (AUROC 0.74).^29^ In a study of community‐dwelling older adults, Lee et al. applied an ML model based on CT SMA and body fat area, age and sex to detect sarcopenia defined using the AWGS 2019 guideline, yielding an AUROC of 0.860.^5^ We observed that the multi‐outcome model developed in this study performed comparably with prior studies in detecting osteoporosis (AUROC 0.864 to 0.911)^29^ and sarcopenia (AUROC 0.847 to 0.871),^5^ whereas detection performance for MS (AUROC 0.755 to 0.845) was comparable with that of skeletal muscle index or visceral adipose tissue alone (AUROC 0.776 to 0.862) in the previous study.^28^ This may be partly due to ethnic differences in relation to visceral fat accumulation and cardiometabolic risk profiles^30^; however, this hypothesis needs to be examined further. Lack of laboratory features in the model may also contribute to the relatively lower performance of the model to detect MS. Although there are several studies for CT body composition models to detect a single disease, we sought to build a multi‐outcome MLP model to detect MS, osteoporosis and sarcopenia, defined according to operational definitions that are widely accepted in current clinical practice by established guidelines, simultaneously from CT scans.

Previous studies showed the prognostic value of CT‐derived body composition for mortality in various populations, including asymptomatic adults and patients with cancer.^31^, ^32^ Our findings support and extend prior studies by providing evidence that metabolic phenotypes (presence of metabolic disorders; MS, osteoporosis and sarcopenia alone or in combination at the individual patient level) detected by a single CT MLP multi‐outcome model using CT body composition features were able to predict mortality in adults aged 50 or older. The association between model‐predicted metabolic phenotypes and mortality remained robust after adjustment for the comorbidity index. These findings indicate the independent prognostic value of CT‐based metabolic phenotyping in addition to conventional measurement of the comorbidity burden using diagnosis codes. Among all metabolic phenotypes, the presence of sarcopenia combined with osteoporosis showed a greater association with mortality compared with other phenotypes, including osteoporosis or sarcopenia alone, which was well aligned with the results of unsupervised clustering analysis. These findings may provide a rationale for the concept of ‘osteosarcopenia’ that has been actively studied recently.^2^

In this study, the age‐related trajectories of multiple CT body composition parameters were plotted. Of note, the unsupervised CT‐based metabolic phenotype clusters revealed distinctive clustering for metabolic disorders with different age distributions. Compared with the youngest middle‐aged population (mean age 50), the youngest old (mean age 65, MS cluster) group had the highest prevalence of MS with visceral fat accumulation, whereas the oldest old (mean age 73, osteosarcopenia cluster) group was characterized by a deficit in skeletal muscle, bone and subcutaneous fat deposits. These findings suggest that CT‐based body composition parameters might serve as imaging biomarkers of metabolic aging, although this hypothesis needs to be further validated.

CT body composition parameters from various levels (L1, L2, L4 or AW) or all levels (incorporating parameters from L1 to L4) yielded similar discriminatory performance for each outcome compared with the results using L3 models. In a study of whole body magnetic resonance imaging of 142 healthy adults aged 19–65 years, a single scan at the level of L3 explained the majority of the variance of the total tissue volumes for skeletal muscle and fat, providing the basis for choosing L3 as a representative level in several practice guidelines for sarcopenia management, including the European Working Group on Sarcopenia in Older People (EWGSOP) 2019.^1^, ^33^

Skeletal muscle radiodensity (the average HU values of all pixels within the skeletal muscle mask) contributed the most to determining the probability of osteoporosis and sarcopenia in this study. Lower skeletal muscle radiodensity is associated with increased fat infiltration in skeletal muscle and intramyocellular lipid contents, which was confirmed by muscle biopsy.^34^ Ectopic fat infiltration in skeletal muscle detected in abdominal CT was associated with impaired physical performance in community‐dwelling older adults and patients with cancer, thus supporting the major contribution of SMD as a predictor for osteoporosis and sarcopenia in our MLP model.^35^ Of note, Pickhardt and colleagues reported that automated CT biomarkers, including SMD, predicted cardiovascular events and overall mortality with a higher discriminatory value than that of clinical anthropometric measurements.^25^ Taken together, these findings suggest that SMD might represent a sensitive imaging biomarker for metabolic alteration that starts to decline at a younger age, reflecting ectopic fat accumulation as a result of metabolic aging.

A distinctive role of visceral fat and subcutaneous fat depots in glucose and bone metabolism has been suggested.^36^, ^37^ In this study, the VFA was the most important contributing feature in the MLP model for detecting MS. Visceral fat accumulation and VFA to SFA are considered core components of the MS, ultimately contributing to insulin resistance.^37^ However, unlike visceral fat, loss of the SFA was associated with a higher probability of osteoporosis and sarcopenia in our model. The SFA is a parameter reported to be beneficial against metabolic diseases, known as the protective fat depot hypothesis.^38^ The SFA is associated with lower triglyceride, glucose and lipid levels.^39^ Our findings well align with the prior notion that visceral fat and subcutaneous fat contribute differentially to the presence of metabolic disorders, which further confirms the model's reliability.

This study has several limitations. As this study used a cross‐sectional dataset, longitudinal changes in body composition at the individual level cannot be inferred. CT image accessibility in low‐resource settings is currently limited and still has radiation exposure issues. Extracted CT body composition parameters, especially the radiodensity of skeletal muscle, could be affected by various CT protocols and scanners,^40^ although CT images were standardized and normalized throughout the entire dataset to improve segmentation and quantification accuracy before running these modules. This study is limited to Korean data; therefore, whether these findings can be applicable to ethnicities other than Korean needs to be further investigated.

In summary, our CT body composition‐based MLP model detected MS, osteoporosis and sarcopenia simultaneously in community‐dwelling and hospitalized adults. Metabolic phenotypes predicted by the CT MLP model were associated with long‐term mortality, which merits further investigation.

## Conflict of interest statement

The authors declare that they have no conflicts of interest.

## Supporting information


**Table S1.** Age‐ and sex‐stratified sampling of participants in derivation cohort and external test cohort.
**Table S2.** Specification for computed tomography scan protocols.
**Table S3.** Hyperparameter of multi‐label deep learning model.
**Table S4.** Summary of CT‐derived multi‐level body composition parameters at abdomen.
**Table S5.** Clinical characteristics of CT body composition‐based metabolic clusters grouped by unsupervised hierarchical clustering in derivation cohort.
**Table S6.** Discriminatory performance of clinical, CT‐derived body composition parameters (L3 level), and combined models for the metabolic syndrome, osteoporosis, and sarcopenia in internal and external test sets.
**Table S7.** Performance of models to detect metabolic syndrome, osteoporosis, or sarcopenia in prior studies.
**Figure S1.** Semi‐automated segmentation of body composition parameters using abdominal computed tomography scans. Abbreviations: AW, WHO‐defined average waist between margin of 12th rib and iliac crest.
**Figure S2.** Architecture of multi‐layer perceptron model for predicting multiple metabolic outcomes using body composition parameters with or without clinical variable. The number of hidden neurons were 2/3 the size of the input layer, plus the size of the output layer with two hidden layers.
**Figure S3.** Age‐dependent trajectory of computed tomography‐derived visceral and subcutaneous fat density in derivation cohort. Abbreviations: AW, average waist.
**Figure S4.** Forest plots of CT‐derived multi‐level body composition parameters at abdomen in internal test set.
**Figure S5.** Forest plots of CT‐derived multi‐level body composition parameters at abdomen in external test set 1.
