# Supplementary material for: Metabolic phenotyping with computed tomography deep learning for metabolic syndrome, osteoporosis and sarcopenia predicts mortality in adults
Source: J Cachexia Sarcopenia Muscle. 2024 Apr 22;15(4):1418–29. doi: 10.1002/jcsm.13487 (PMC11294037; doi:10.1002/jcsm.13487)
Supplement: Supplementary file 1 — Table S1. Age‐ and sex‐stratified sampling of participants in derivation cohort and external test cohort. Table S2. Specification for computed tomography scan protocols. Table S3. Hyperparameter of multi‐label deep learning model. Table S4. Summary of CT‐derived multi‐level body composition parameters at abdomen. Table S5. Clinical characteristics of CT body composition‐based metabolic clusters grouped by unsupervised hierarchical clustering in derivation cohort. Table S6. Discriminatory performance of clinical, CT‐derived body composition parameters (L3 level), and combined models for the metabolic syndrome, osteoporosis, and sarcopenia in internal and external test sets. Table S7. Performance of models to detect metabolic syndrome, osteoporosis, or sarcopenia in prior studies. Figure S1. Semi‐automated segmentation of body composition parameters using abdominal computed tomography scans. Abbreviations: AW, WHO‐defined average waist between margin of 12th rib and iliac crest. Figure S2. Architecture of multi‐layer perceptron model for predicting multiple metabolic outcomes using body composition parameters with or without clinical variable. The number of hidden neurons were 2/3 the size of the input layer, plus the size of the output layer with two hidden layers. Figure S3. Age‐dependent trajectory of computed tomography‐derived visceral and subcutaneous fat density in derivation cohort. Abbreviations: AW, average waist. Figure S4. Forest plots of CT‐derived multi‐level body composition parameters at abdomen in internal test set. Figure S5. Forest plots of CT‐derived multi‐level body composition parameters at abdomen in external test set 1. [file JCSM-15-1418-s001.pdf]

[Supplementary Materials]

Metabolic phenotyping with CT deep learning for metabolic syndrome, osteoporosis, and sarcopenia predicts mortality in adults [1]

Sang Wouk Cho[1,2*], Seungjin Baek[1*], SooKyoung Han[1], Chang Oh Kim[3], Hyeon Chang Kim[4], Yumie Rhee[1], Namki Hong[1]

[1]Department of Internal Medicine, Endocrine Research Institute, Severance Hospital, Yonsei University College of Medicine, Seoul, Korea

[2]Department of Integrative Medicine, Yonsei University College of Medicine, Seoul, Korea

[3]Division of Geriatric Medicine, Department of Internal Medicine, Yonsei University College of Medicine, Seoul, Korea

[4]Department of Preventive Medicine, Yonsei University College of Medicine, Seoul, Korea

*These two authors contributed equally to this work.

Corresponding author: Namki Hong, M.D., M.P.H., Ph.D.

Department of Internal Medicine, Yonsei University College of Medicine,

50-1 Yonsei-ro, seodaemun-gu, Seoul, South Korea (03722)

Email: nkhong84@yuhs.ac; office: 02-2228-0790

---

[1] *Abbreviations:* CT, computed tomography; MS, metabolic syndrome; SHAP, SHapley Additive exPlanations; MLP, multi-layer perceptron; SMD, skeletal muscle density; BD, bone density; SMA, skeletal muscle area; VFA, visceral fat area; BMI, body mass index; CMERC, Cardiovascular and Metabolic Diseases Etiology Research Center; KURE, Korean Urban Rural Elderly; BMD, bone mineral density; QCT, quantitative computed tomography; VFD, visceral fat density: SFA, subcutaneous fat area; SFD, subcutaneous fat density.

Contents

Supplementary table 1. Age- and sex-stratified sampling of participants in derivation cohort and external test cohort

Supplementary table 2. Specification for computed tomography scan protocols

Supplementary table 3. Hyperparameter of multi-label deep learning model

Supplementary table 4. Summary of CT-derived multi-level body composition parameters at abdomen

Supplementary table 5. Clinical characteristics of CT body composition-based metabolic clusters grouped by unsupervised hierarchical clustering in derivation cohort

Supplementary Table 6. Discriminatory performance of clinical, CT-derived body composition parameters (L3 level), and combined models for the metabolic syndrome, osteoporosis, and sarcopenia in internal and external test sets

Supplementary Table 7. Performance of models to detect metabolic syndrome, osteoporosis, or sarcopenia in prior studies

Supplementary figure 1. Semi-automated segmentation of body composition parameters using abdominal computed tomography scans. Abbereviations: AW, WHO-defined average waist between margin of 12th rib and iliac crest.

Supplementary figure 2. Architecture of multi-layer perceptron model for predicting multiple metabolic outcomes using body composition parameters with or without clinical variable. The number of hidden neurons were 2/3 the size of the input layer, plus the size of the output layer with two hidden layers.

Supplementary figure 3. Age-dependent trajectory of computed tomography-derived visceral and subcutaneous fat density in derivation cohort. Abbreviations: AW, average waist

Supplementary figure 4. Forest plots of CT-derived multi-level body composition parameters at abdomen in internal test set

Supplementary figure 5. Forest plots of CT-derived multi-level body composition parameters at abdomen in external test set 1

References for supplementary material

Supplementary table 1. Age- and sex-stratified sampling of participants in derivation cohort and external test cohort

	Derivation cohort (n=516)		External test cohort (n=380)	
Age groups	Women	Men	Women	Men
35-39	20	17	-	-
40-49	50	40	46	49
50-59	49	50	48	46
60-69	48	48	47	49
70-79	48	50	49	46
80 or older	49	47	-	-

Supplementary table 2. Specification for computed tomography scan protocols

	Derivation cohort (n=516)		External test cohort 1 (n=380)
Dataset	CMERC (n=289)	KURE (n=227)	Severance check-up
kVp	120	100	100
Slice thickness	3 mm	3 mm	3 mm
FOV	500	500	500
Kernel	B40s<br>(n=235, 81.3%)<br>Standard<br>(n=54, 18.7%)	B30f<br>(n=227, 100%)	Standard<br>(n=190, 50%)<br>Soft<br>(n=190, 50%)
Manufacturers	SIEMENS<br>(SOMATOM Definition AS+<br>[n=14, 4.8%];<br>Sensation 64<br>[n=221, 76.5%])<br>GE medical systems<br>(LightSpeedVCT<br>[n=54/18.7%])	SIEMENS<br>(SOMATOM<br>Definition AS+<br>[n=89, 39.2%],<br>SOMATOM<br>Definition Flash<br>[n=138, 60.8%])	GE medical systems<br>(LightSpeedVCT<br>[n=380, 100%])
	External test cohort 2 (n=10,141)		
Dataset	Severance Hospital		
kVp	<100(<1%); 100 (34%); 110(<1%); 120(66%); >120(<1%)		
Slice thickness	< 3mm (2%); 3mm (88%); >3mm (10%);		
FOV	500		
Kernel	Standard (37%); Soft (14%);B40s (20%); B30f (n=906, 9%)<br>B31f (8%); Other (12%)		
Manufacturers	GE medical systems<br>- LightSpeed Plus [<1%]<br>- LightSpeed VCT [37%]<br>- Revolution CT [3%]<br>- Revolution EVO [9%]<br>- Discovery CT750 HD [2%]<br>SIEMENS<br>- Sensation 16 [<1%]<br>- Sensation 64 [13%]<br>- SOMATOM Definition AS+ [22%]<br>- SOMATOM Definition Flash [7%]<br>- SOMATOM Force [2%]<br>- SOMATOM X.cite [<1%]<br>Philips<br>- iCT 256 [4%]<br>- Ingenuity CT [<1%]<br>Cannon<br>- Aquilion [n4,<1%]<br>- Aquilion ONE [<1%]		

Supplementary table 3. Hyperparameter of multi-label deep learning model

Parameters type	Training parameters	
Training parameters	Learning rate	0.001 - 0.002 (Adjusted by epoch)
	Optimizer	Adam
	Loss function	Multi-label binary cross-entropy
	Activation function	Relu
	Bias	True
Hardware	CPU	Intel(R) Xeon(R) Silver 4210 CPU @ 2.20GHz
	GPU	Nvidia GeForce RTX 4080 Graphics Cards

Supplementary table 4. Summary of CT-derived multi-level body composition parameters at abdomen

Level	Age	VFA, cm^2		SFA, cm^2		SMA, cm^2		SMD, HU		BD, HU	
		Mean	SD	Mean	SD	Mean	SD	Mean	SD	Mean	SD
Women											
L1	30-39	24	21	80	52	84	13	42	4	365	28
	40-49	41	28	96	49	82	13	37	5	352	36
	50-59	72	40	114	33	84	13	33	5	288	41
	60-69	92	51	137	48	84	12	26	7	278	43
	70-79	107	60	154	64	84	15	17	8	253	48
	≥80	92	49	118	48	75	12	18	7	232	35
L2	30-39	28	56	105	61	95	13	41	4	404	42
	40-49	44	27	121	56	92	14	37	4	378	38
	50-59	74	42	137	38	93	15	33	5	316	40
	60-69	99	52	160	57	92	12	26	8	295	44
	70-79	121	63	176	66	92	16	17	8	269	53
	≥80	111	57	138	53	83	11	17	7	242	37
L3	30-39	33	28	128	64	109	12	43	4	424	31
	40-49	50	28	146	63	103	14	39	5	395	41
	50-59	77	43	155	43	102	16	35	5	332	43
	60-69	100	49	180	57	100	13	27	8	307	48
	70-79	122	60	194	75	98	15	18	9	286	55
	≥80	119	56	161	58	90	12	17	7	257	42
L4	30-39	30	19	158	71	107	9	41	4	425	38
	40-49	48	23	176	66	103	12	38	4	402	50
	50-59	68	33	187	47	100	15	35	4	332	49
	60-69	89	39	210	64	96	14	26	8	317	96
	70-79	110	49	221	75	90	13	17	8	307	95
	≥80	117	50	191	68	86	14	16	7	263	47
AW	30-39	27	21	111	58	91	11	42	4	362	25
	40-49	43	25	125	51	88	12	38	4	342	31
	50-59	69	36	140	35	88	13	34	5	284	35
	60-69	89	43	161	51	87	11	27	7	269	41
	70-79	105	50	175	64	86	13	18	7	252	45
	≥80	98	46	146	52	78	11	17	6	230	31
Men											
L1	30-39	123	59	95	35	136	21	43	4	310	33
	40-49	124	60	94	47	130	15	42	5	317	36
	50-59	122	56	69	27	119	18	41	4	297	48
	60-69	141	57	78	30	116	15	36	6	291	54
	70-79	136	81	71	30	105	17	30	6	286	49
	≥80	123	65	66	27	95	15	28	6	274	68
L2	30-39	127	56	119	41	153	22	44	4	342	57
	40-49	130	54	120	60	150	18	43	5	346	39
	50-59	123	54	89	34	134	21	41	4	325	48
	60-69	146	59	97	35	130	18	37	6	320	66
	70-79	151	85	89	37	119	18	29	6	312	66

	≥80	137	69	83	31	109	15	27	5	301	70
L3	30-39	102	47	153	48	170	21	46	4	342	57
	40-49	115	47	155	73	165	17	44	5	346	39
	50-59	113	51	116	43	151	21	42	4	325	48
	60-69	132	57	125	46	145	18	38	6	320	66
	70-79	142	81	116	45	132	19	60	6	312	66
	≥80	132	69	106	35	120	16	28	6	301	70
L4	30-39	78	35	188	57	155	15	45	4	371	52
	40-49	86	35	187	78	154	20	44	5	388	51
	50-59	82	33	142	50	137	20	42	4	356	55
	60-69	98	46	156	55	130	18	37	6	357	62
	70-79	113	56	143	51	118	18	30	6	350	65
	≥80	106	56	128	41	109	14	28	6	335	77
AW	30-39	107	49	127	40	144	18	44	4	305	33
	40-49	110	48	128	58	139	15	43	5	315	30
	50-59	105	45	96	34	126	17	41	4	293	41
	60-69	124	50	106	37	123	15	37	6	292	49
	70-79	120	67	98	37	110	17	30	6	287	48
	≥80	111	57	91	30	100	13	28	5	274	52

Abbreviations: VFA, visceral fat area; SFA, subcutaneous fat area; SMA, skeletal muscle area; SMD, skeletal muscle density; BD, bone density; HU, Hounsfield unit; AW, World Health Organization-defined average waist as middle line between 12th rib margin and iliac crest.

Supplementary table 5. Clinical characteristics of CT body composition-based metabolic clusters grouped by unsupervised hierarchical clustering in derivation cohort

*: P<0.05 vs. cluster 1 (normal cluster, reference)

	Cluster 1 (Normal) n=151	Cluster 2 (Metabolic syndrome) n=230	Cluster 3 (Osteosarcopenia) n=135	p-value
Age, year	49.7 ± 9.0	65.8 ± 13.6*	72.3 ± 10.9*†	<0.001
Women, n(%)	85 (56)	121 (53)	58 (43)	0.067
L3 VFA, cm^2	67.2 ± 37.1	151.3 ± 53.2*	76.1 ± 46.4†	<0.001
L3 VFD, HU	-93.8 ± 6.6	-102.2 ± 4.1*	-87.8 ± 7.9*†	<0.001
L3 SFA, cm^2	120.9 ± 37.7	182.0 ± 61.6*	107.7 ± 41.3*†	<0.001
L3 SFD, HU	-98.6 ± 4.8	-104.9 ± 4.6*	-96.4 ± 8.4*†	<0.001
L3 SMA, cm^2	124.5 ± 28.4	125.9 ± 32.5	108.6 ± 22.9*†	<0.001
L3 SMD, HU	42.3 ± 5.0	28.8 ± 10.6*	29.3 ± 10.4*	<0.001
L3 BD, HU	382.9 ± 50.3	323.6 ± 58.6*	296.7 ± 54.7*	<0.001
Metabolic syndrome, n(%)	14 (9)	89 (39)	13 (10)	<0.001
Sarcopenia, n(%)	3 (2)	12 (5)	36 (27)	<0.001
Osteoporosis, n(%)	3 (2)	72 (30)	68 (50)	<0.001

†: P<0.05 vs. cluster 2 (metabolic syndrome cluster)

Abbreviations: VFA, visceral fat area; VFD, visceral fat density; SFA, subcutaneous fat area; SFD, subcutaneous fat density; SMA, skeletal muscle area; SMD, skeletal muscle density; BD, bone density.

Supplementary Table 6. Discriminatory performance of clinical, CT-derived body composition parameters (L3 level), and combined models for the metabolic syndrome, osteoporosis, and sarcopenia in internal and external test sets

	L3*			L3+clinical†		
	Metabolic syndrome	Osteoporosis	Sarcopenia	Metabolic syndrome	Osteoporosis	Sarcopenia
Internal test set (n=129)						
AUROC	0.827	0.916	0.856	0.845	0.911	0.871
AUPRC	0.569	0.847	0.535	0.538	0.780	0.631
Accuracy	0.789	0.859	0.719	0.773	0.820	0.766
Precision	0.513	0.707	0.227	0.490	0.615	0.250
Recall	0.714	0.829	0.833	0.857	0.914	0.750
F1 score	0.597	0.763	0.357	0.623	0.736	0.375
External test set (n=380)						
AUROC	0.728	0.883	0.777	0.755	0.897	0.847
AUPRC	0.407	0.689	0.543	0.453	0.751	0.602
Accuracy	0.737	0.863	0.761	0.721	0.892	0.837
Precision	0.455	0.628	0.416	0.437	0.696	0.591
Recall	0.495	0.681	0.568	0.571	0.764	0.527
F1 score	0.474	0.653	0.480	0.495	0.728	0.557
Threshold	0.272	0.400	0.064	0.2596	0.2291	0.0149

*DNN model based on L3 level body composition parameters as input features

†DNN model based on L3 level body composition parameters and clinical variables (age, sex, BMI) as input features

Abbreviations: AUROC, area under the receiver-operating characteristics curve; AUPRC, area under the precision-recall curve

Supplementary Table 7. Performance of models to detect metabolic syndrome, osteoporosis, or sarcopenia in prior studies

First author	References	Study population	Model performance (AUROC)		
			Metabolic syndrome	Osteoporosis	Sarcopenia
Pickhardt	[1] AJR Am J Roentgenol. 2021 Jan; 216(1):85-92.	N=7785 (women 4361; mean age 57 years)	Prevalence: 9.5%<br><br>Total abdominal tissue + skeletal muscle index: 0.916<br>Visceral adipose tissue: 0.862<br>Skeletal muscle index: 0.776	Not applicable	Not applicable
Vadera	[2] Insights Imaging . 2023 Apr 1;14(1):57.	N=536 (women 394; 44% osteoporosis defined using DXA T-score; mean age 65.8 years)	Not applicable	Prevalence: 44% (defined using DXA T-score)<br><br>L1 attenuation: 0.74	Not applicable
Lee	[3] Clin Nutr. 2021 Aug;40(8):5038-5046.	N=522 (women 352; mean age 75 years)	Not applicable	Not applicable	Prevalence: 16% defined by AWGS 2019 definition<br><br>CT SMA + body fat area: 0.810<br>CT SMA + body fat area + age + sex: 0.860
Cho	This study	N=516 (internal test set n=129, women 52%, mean age 62 years); external test set n=380, women 50%, mean age 59 years	Internal test set: 22%<br>CT alone: 0.827<br>CT+Clinical: 0.845<br><br>External test set: 24%<br>CT alone: 0.728<br>CT + clinical: 0.755	Internal test set: 22%<br>CT alone: 0.916<br>CT+Clinical: 0.911<br><br>External test set: 24%<br>CT alone: 0.883<br>CT + clinical: 0.897	Internal test set: 22%<br>CT alone: 0.856<br>CT+Clinical: 0.871<br><br>External test set: 24%<br>CT alone: 0.777<br>CT + clinical: 0.847

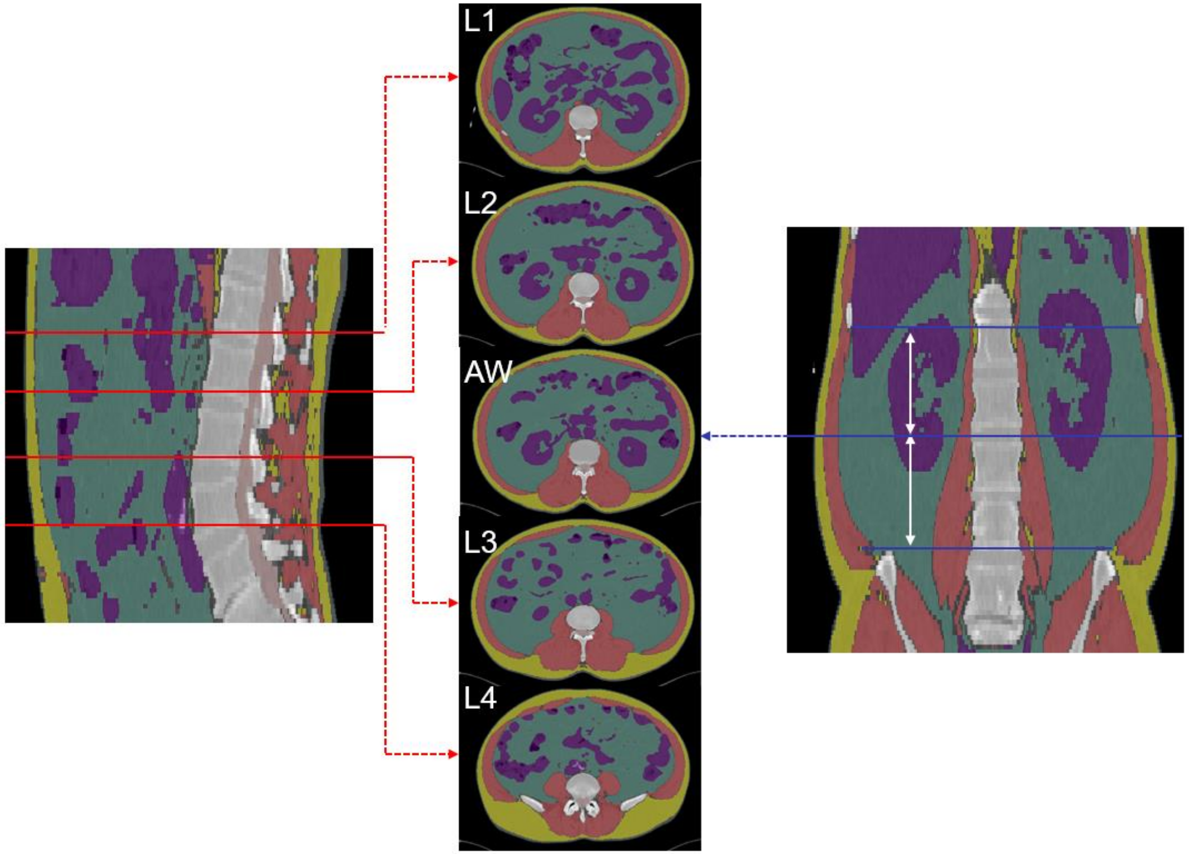

Supplementary figure 1. Semi-automated segmentation of body composition parameters using abdominal computed tomography scans. Abbereviations: AW, WHO-defined average waist between margin of 12th rib and iliac crest.

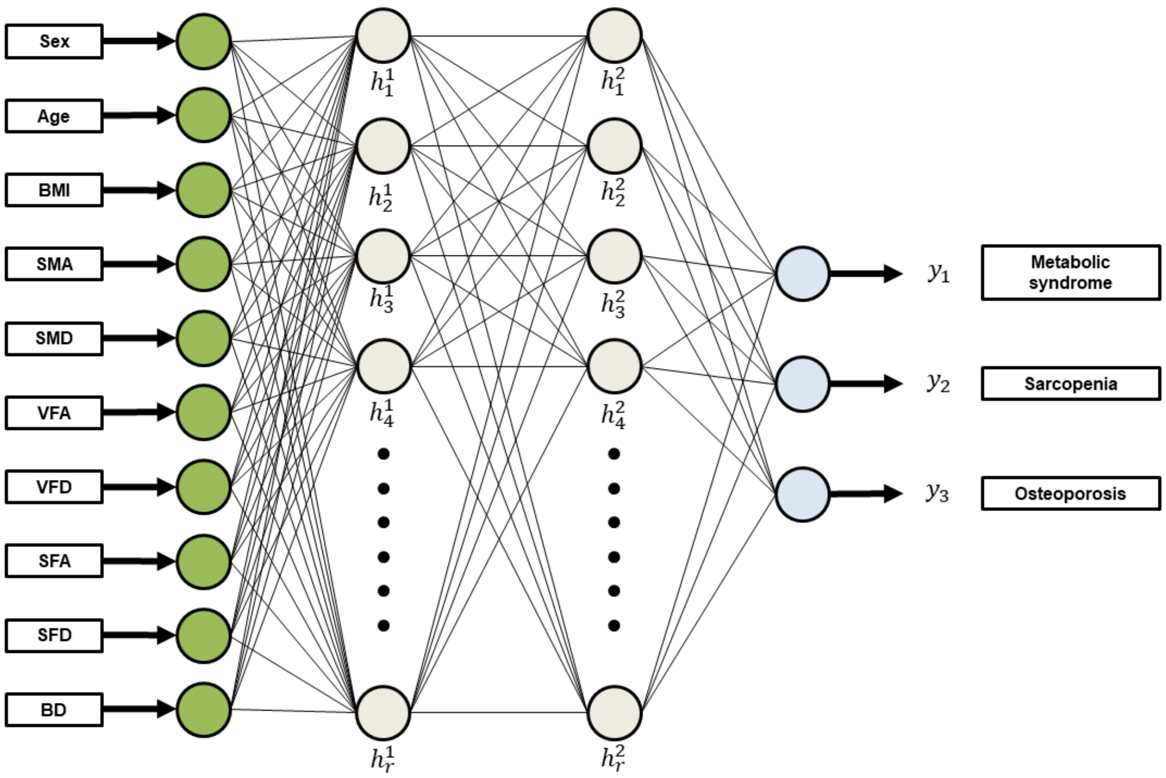

Supplementary figure 2. Architecture of multi-layer perceptron model for predicting multiple metabolic outcomes using body composition parameters with or without clinical variable. The number of hidden neurons were 2/3 the size of the input layer, plus the size of the output layer with two hidden layers.

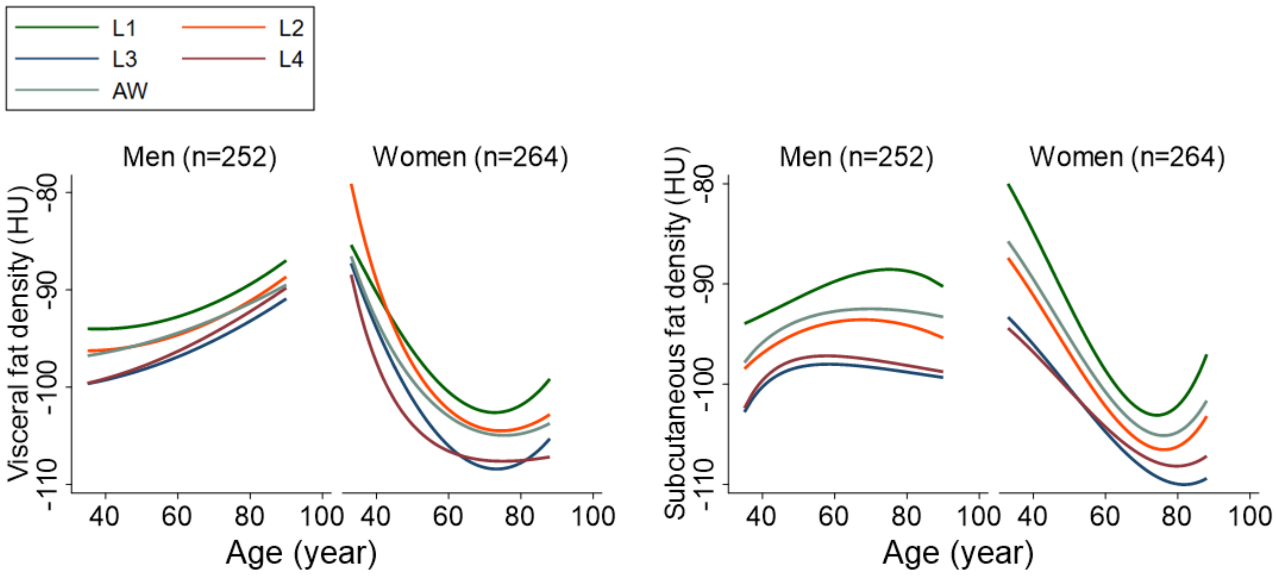

Supplementary figure 3. Age-dependent trajectory of computed tomography-derived visceral and subcutaneous fat density in derivation cohort. Abbreviations: AW, average waist

Supplementary figure 4. Forest plots of CT-derived multi-level body composition parameters at abdomen in internal test set

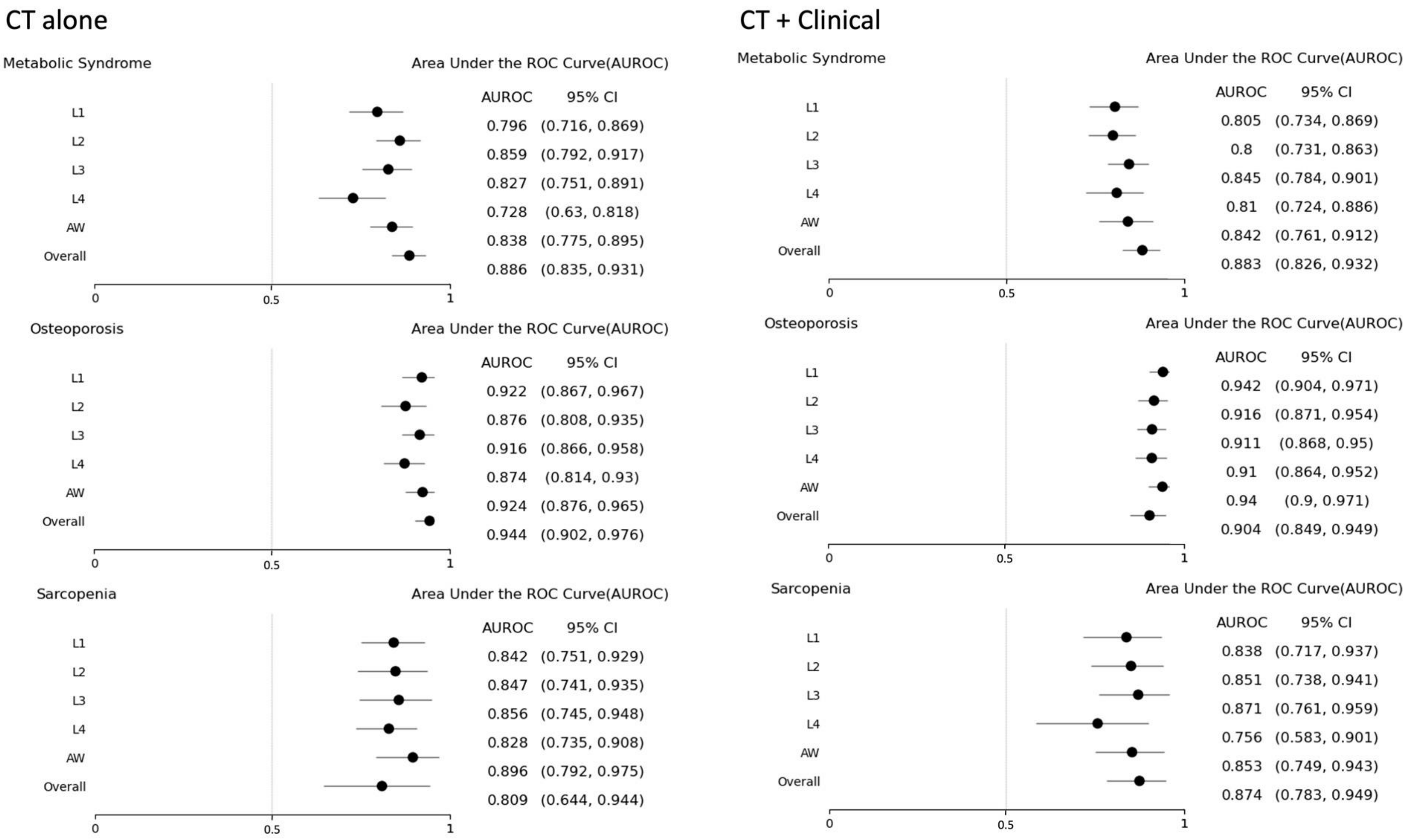

Supplementary figure 5. Forest plots of CT-derived multi-level body composition parameters at abdomen in external test set 1

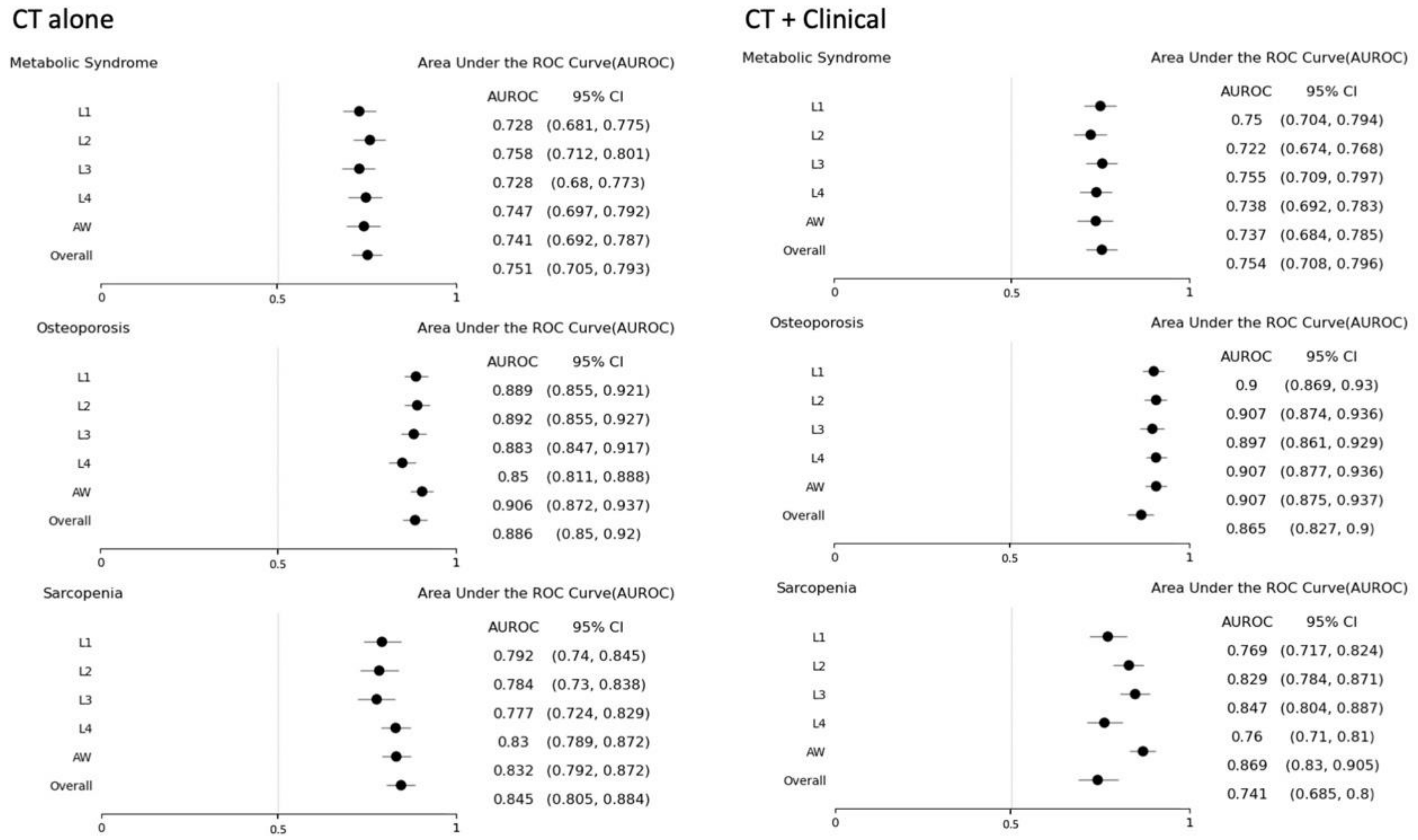

References for supplementary material

1. Pickhardt PJ, Graffy PM, Zea R, Lee SJ, Liu J, Sandfort V, Summers RM (2021) Utilizing Fully Automated Abdominal CT-Based Biomarkers for Opportunistic Screening for Metabolic Syndrome in Adults Without Symptoms. AJR Am J Roentgenol 216:85-92
2. Vadera S, Osborne T, Shah V, Stephenson JA (2023) Opportunistic screening for osteoporosis by abdominal CT in a British population. Insights Imaging 14:57
3. Lee YS, Hong N, Witanto JN, Choi YR, Park J, Decazes P, Eude F, Kim CO, Chang Kim H, Goo JM, Rhee Y, Yoon SH (2021) Deep neural network for automatic volumetric segmentation of whole-body CT images for body composition assessment. Clin Nutr 40:5038-5046
